# Co-occurrence of Multiple Sclerosis and Severe Aplastic Anemia: A Report of Two Cases Successfully Treated with Allogeneic Hematopoietic Stem Cell Transplantation

**DOI:** 10.1007/s44228-023-00028-8

**Published:** 2023-02-17

**Authors:** Alfadil Haroon, Syed Osman Ahmed, Mahmoud Aljurf, Etedal AbuElbasher, Hazzaa Alzahrani

**Affiliations:** 1 Oncology Centre, King Faisal Specialist Hospital and Research Centre, Section of Adult Hematology/HSCT, PO Box 3354, Riyadh, 11471 Saudi Arabia; 2 Alneelin University, National Centre for Neurological Science, Khartoum, Sudan

**Keywords:** Multiple Sclerosis, Allogeneic Hematopoietic Stem Cell transplantation, Severe aplastic anemia, Conditioning regiment and stem cell boost

Multiple Sclerosis (MS) is an autoimmune disease believed to be secondary to T cells autoreactive against myelin, leading to demyelination, neurodegeneration and, eventually, disability [1]. Four subtypes are described, depending on the behavior of the disease: relapsing–remitting, primary progressive, secondary progressive and relapsing progressive. The management is mainly based on disease modifying therapies (DMT) and steroids for acute attacks. Many DMT are available in clinical practice, including old (interferon β, alemtuzumab and glatiramer acetate) [2], and novel agents (natalizumab, daclizumab, ocrelizumab, etc.). The focus of these treatments is to decrease progression and minimize or delay the disability [2]. Despite the availability of these drugs, many patients progress and succumb to their disease. Autologous hematopoietic stem cell transplantation (ASCT) seems to be beneficial to patients in the relapsing–remitting with high inflammatory activity subgroup after failing the standard of care DMTs, and to those with aggressive MS who had developed severe disability in the prior 12 months [3]. Aplastic anemia (AA) is a form of bone marrow failure which is usually idiopathic, but can also develop because of infections, drugs (some are MS drugs) among other etiologies [4–6]. The treatment of AA depends on the severity of the disease and the age of the patient. Allogeneic Hematopoietic Stem Cell transplantation (allo-SCT) is the standard of care for young patients with severe AA [4]. We herein present two MS patients, who were allotransplanted for severe AA (Table 1).

**Table 1:** Patients’ characteristics

		Case 1	Case 2
1	Age	45	42
2	Gender	Male	Female
3	Hematological Diagnosis	SAA	SAA
4	Karyotype	Normal	Normal
5	PNH	Small clone	Small clone
6	Patient HLA-DRB1 genotype	DRB1*03:01	DRB1*15:01
7	Donor type	MSD (male donor)	MSD (female donor)
8	Stem cell source	Bone marrow	PBSC
9	Conditioning	1st HSCT FLU/CY 2nd HSCT FLU/CY/Alemtuzumab	Flu/Cy/ATG/TBI 200
10	Stem cell dose (CD34)	1st HSCT 1.46 × 10^6^/kg 2nd HSCT 3.2 × 10^6^/kg	5.04 X10^6^/kg
11	GvHD prophylaxis	MTX/CSA	MTX/CSA
12	Chimerism	Poor donor-lymphocyte engraftment improved after mega dose of stem cell boost	100%
13	Graft failure	Yes 2 times	No
14	GvHD	Grade 1 liver GvHD post DLI	No GvHD
15	Duration from MS Diagnosis to SAA	9 months	5 years
16	MS type	Progressive relapsing MS	Relapsing remitting
17	Neurological signs and symptoms	Parapresis with sensory level	Parapresis with sensory level
18	Diagnostic MRI lesions pre- HSCT	Corpus callosum, and deep white matter spinal cord lesions	Supra and infra tentorial and spinal cord lesions
19	MS Treatment before HSCT	IFN-β 1a	IFN-β 1a
20	MS Treatment response	Poor response	Good response
21	MS course Post HCT	Slow progression	Stable disease
22	Residual disabilities	Yes	No
23	Interval from last Interferon dose to SAA development	One month	One month
24	EDSS Post HSCT	6	4.5

*MRI* magnetic resonance imaging, *TBI* total body irradiation, *PBSC* peripheral blood stem cells, *MS* Multiple Sclerosis, *HSCT* hematopoietic stem cell transplantation, *GvHD* graft versus host disease, *EDSS* Expanded Disability Status Scale, *ATG* anti-thymocyte globulin

A 45-year-old man was diagnosed with severe AA in April 2014, after presenting with ecchymosis and epistaxis. His workup showed pancytopenia (ANC of 0.49 × 10^9^/L, platelets of 10 × 10^9^/L, hemoglobin of 6 g/dl) with 5% cellularity on the bone marrow biopsy. He underwent allo-SCT from his matched brother using fludarabine/cyclophosphamide (flu/cy) conditioning and bone marrow as a stem cell source. His course was complicated by mixed lymphoid chimerism. His GvHD prophylaxis consisted of short methotrexate (MTX) course and cyclosporine (CSA). Nine months post-transplant CSA tapering was started. However, 6 weeks after tapering he developed unsteady gait and, after an extensive workup, he was diagnosed with MS, based on the detection of typical MRI T2 lesions in periventricular, cortical and infratentorial areas of the brain, (Fig. 1) and oligoclonal bands in the CSF.

**Fig. 1:**
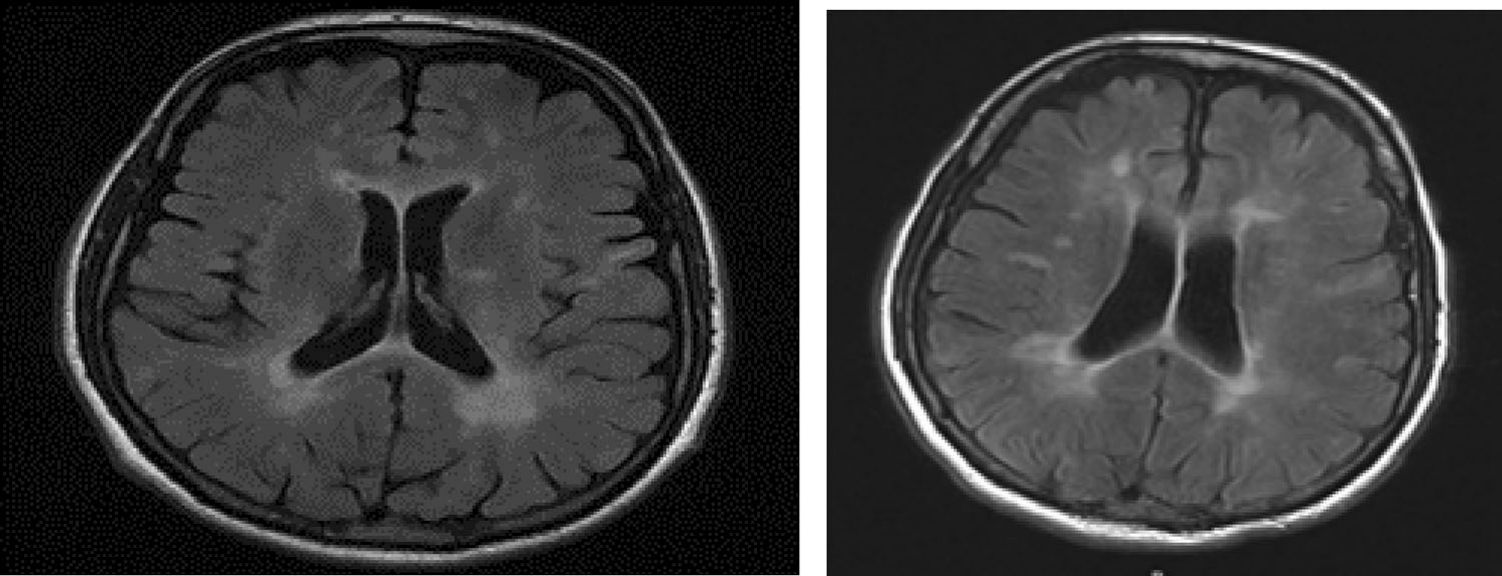
MRI brain showing T2 and FLAIR Abnormal signal hyperintensity in deep white matter tracts cerebral parenchyma—case 1

After neurological evaluation and an initial observation phase, he was started on interferon beta-1a (IFN-β 1a). However, a month after the first dose, he developed pancytopenia, and an extensive workup showed hypocellular bone marrow (5%) with full donor chimerism (infectious, nutritional deficiencies, and autoimmune causes were ruled out). Simultaneously, his MS symptoms got worse, the expanded disability status scale (EDSS) was 6.5 and an MRI showed new spinal cord demyelinating lesions. He then underwent a second allo-SCT from the same donor using FCC conditioning and mycofenolate mofetil (MMF) for GvHD prophylaxis. He engrafted by day 14 and his sensory symptoms improved as well.

His second post-transplant course was complicated by poor graft function and mixed chimerism (Fig. 2) which eventually led to graft failure that did not respond to immune manipulation and donor lymphocyte infusion (DLI). Ten months after his second transplant he received a stem cell mega dose (total 22.11 × 10^8^/kg nucleated cells infused, including 11.25 × 10^6^/kg CD34 stem cells and 39.98 × 10^7^/kg CD3 + /kg). Currently, 56 months after his stem cell boost, he remains in remission of the severe AA, and his MS is stable, with an EDSS of 6, and no further clinical or radiological progression.

**Fig. 2:**
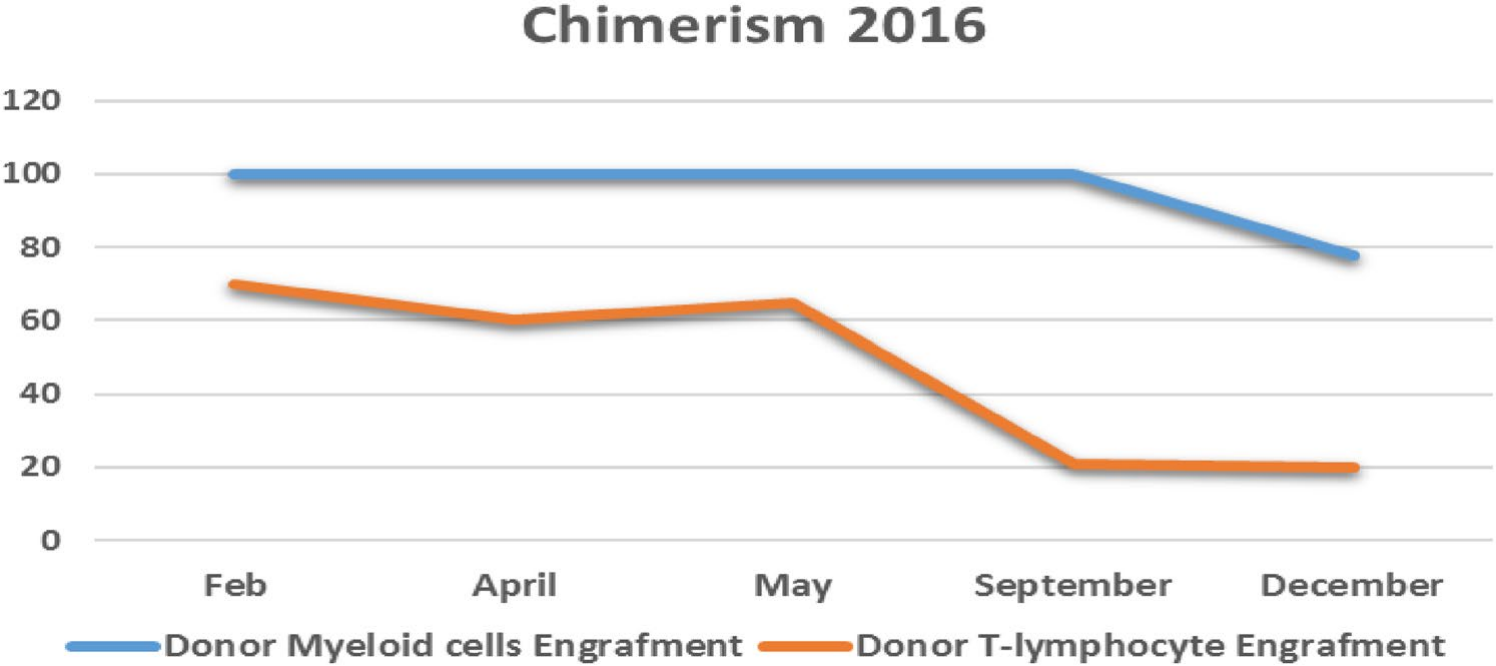
Showing chimerism post second HSCT—case 1

A 42-year-old woman, diagnosed with MS in 2014, started on IFN-β 1a with good control, and no further relapses. In 2019, she was diagnosed with AA, after presenting with vaginal bleeding, and was found to have pancytopenia (WBC 3.49 × 10^9^/L, HGB 81 g/L ANC 0.33 × 10^9^/L, platelet 3 × 10^9^/L). IFN-β 1a was suspended, and she was started on horse ATG at 40 mg/m^2^ and CSA, with no improvement. She then underwent matched sibling donor allo-SCT in 2020, with Flu/Cy/ATG/TBI conditioning. She engrafted quickly with full donor chimerism and, currently, 27 months post allo-SCT, she is off immunosuppression, with no further progression of her MS, despite being off MS medications since the AA diagnosis.

The association of MS and SAA has been reported in the literature [7]. The HLA Class II DRB1 antigen DR15 (common alleles *1501, *1502) is observed with a frequency between 20 and 30% in various ethnic populations [8], and has been implicated in the pathogenesis of both AA and MS. Allo-SCT is a standard intervention for young AA patients with a matched sibling, and for the majority of the other AA patients after failing immunosuppressive therapy (IST), even in the absence of a matched sibling donor. Auto-HSCT is a standard option for patients with RRMS after failing one line of DMT [3]. Auto-HSCT is also considered an option, even before failing a full course of DMT, for patients with aggressive RRMS who developed severe disability in the preceding 12 months [3]. It is now considered as a standard, whereas allo-HSCT is generally not recommended or performed only in selected highly refractory cases with an HLA identical sibling donor [9]. Allo-HSCT for MS is rarely performed. GvHD associated with allo-HSCT usually affects the skin, gut, and liver, and only rarely the CNS, in which case there is often a significant systemic GvHD elsewhere [10]. CNS involvement of GvHD is controversial, especially since clinical manifestations of CNS GvHD are heterogeneous [11–14]. Patients with chronic GvHD affecting the CNS may present with stroke-like episodes, MS, transverse myelitis, or acute disseminated encephalomyelitis-like disorders, encephalitis, and other nonspecific neurological symptoms [15]. MS-like relapsing–remitting disease has been reported at ten months after allogeneic HSCT [16]. In the absence of GvHD affecting other organs, that presentation fulfilled neither the international consensus diagnostic criteria for neuromyelitis optica spectrum disorder nor the Grauer et al. criteria for the CNS manifestations of GvHD [17, 18].

Our cases highlight the association of MS and AA. These cases also highlight the issue of CNS GvHD and how to differentiate this from MS.

## Data Availability

Raw data were generated at King Faisal specialist hospital. Derived data supporting the findings of this study are available from the corresponding author [Alfadil,Haroon] on request.

## References

[1] Villar LM (2010). Immunological mechanisms that associate with oligoclonal IgM band synthesis in multiple sclerosis. Clin Immunol.

[2] Gajofatto A, Benedetti MD (2015). Treatment strategies for multiple sclerosis: when to start, when to change, when to stop?. World J Clin Cases.

[3] Bertolotto A (2020). Autologous hematopoietic stem cell transplantation (AHSCT): standard of care for relapsing–remitting multiple sclerosis patients. Neurol Ther.

[4] Killick SB (2016). Guidelines for the diagnosis and management of adult aplastic anaemia. Br J Haematol.

[5] Aslam AK, Singh TJ (2002). Aplastic anemia associated with interferon β-1a. Am J Ther.

[6] Ioannou S (2010). Aplastic anemia associated with interferon alpha 2a in a patient with chronic hepatitis C virus infection: a case report. J Med Case reports.

[7] Hinterberger-Fischer M (1994). Coincidence of severe aplastic anaemia with multiple sclerosis or thyroid disorders. Acta Haematol.

[8] Sugimori C (2007). Roles of DRB1∗ 1501 and DRB1∗ 1502 in the pathogenesis of aplastic anemia. Exp Hematol.

[9] Sharrack B (2020). European Society for Blood and Marrow Transplantation (EBMT) Autoimmune Diseases Working Party (ADWP) and the Joint Accreditation Committee of the International Society for Cellular Therapy (ISCT) and EBMT (JACIE). Autologous haematopoietic stem cell transplantation and other cellular therapy in multiple sclerosis and immune-mediated neurological diseases: updated guidelines and recommendations from the EBMT Autoimmune Diseases Working Party (ADWP) and the Joint Accreditation Committee of EBMT and ISCT (JACIE). Bone Marrow Transplant.

[10] Avivi I (2014). Development of multifocal leukoencephalopathy in patients undergoing allogeneic stem cell transplantation—can preemptive detection of John Cunningham virus be useful?. Int J Infect Dis.

[11] Padovan CS (1999). Angiitis of the central nervous system after allogeneic bone marrow transplantation?. Stroke.

[12] Campbell JN, Morris PP (2005). Cerebral vasculitis in graft-versus-host disease: a case report. Am J Neuroradiol.

[13] Takatsuka H (2000). New imaging findings in a patient with central nervous system dysfunction after bone marrow transplantation. Acta Haematol.

[14] Sostak P (2004). Cerebral endothelial expression of adhesion molecules in mice with chronic graft-versus-host disease. Stroke.

[15] Ruggiu M (2017). Case report: central nervous system involvement of human graft versus host disease: report of 7 cases and a review of literature. Medicine.

[16] Das J (2020). A case of multiple sclerosis—like relapsing remitting encephalomyelitis following allogeneic hematopoietic stem cell transplantation and a review of the published literature. Front Immunol.

[17] Grauer O (2010). Neurological manifestations of chronic graft-versus-host disease after allogeneic haematopoietic stem cell transplantation: report from the Consensus Conference on Clinical Practice in chronic graft-versus-host disease. Brain.

[18] Wingerchuk DM (2015). International consensus diagnostic criteria for neuromyelitis optica spectrum disorders. Neurology.

